# Supplementary material for: Adaptation to sorbic acid in low sugar promotes resistance of yeast to the preservative
Source: Heliyon. 2023 Nov 4;9(11):e22057. doi: 10.1016/j.heliyon.2023.e22057 (PMC10682675; doi:10.1016/j.heliyon.2023.e22057)
Supplement: Multimedia component 1 [file mmc1.docx]

**Supplementary Data**

**Figure S1 – Relative sorbic acid resistance at low versus high glucose is evident over a range of sorbic acid concentrations.**

Growth data for *S. cerevisiae* with varying concentrations of sorbic acid (SA) are presented as the ratio of optical density in 0.1% versus 2% glucose cultures at each indicated time point of growth, where a ratio above 1.0 represents greater OD_600_ in the low glucose condition. Points represent the means from three biological replicates; error bars represent SEM of three biological replicates.

**Figure S2 – Intracellular pH of cells at different glucose levels, treated with weak acids**

Intracellular pH of *S. cerevisiae* after 4 h incubation in YEP supplemented with either 2% or 0.1% glucose in the presence or absence of WAs (supplied at ~55% of their MICs). Error bars represent SEM from three biological replicates, with each replicate value representing the median pH of 10^5^ cells.. ***, p < 0.001, according to one-way ANOVA; ns, not significant.

**Figure S3 – Absence of sorbic acid resistance at low glucose in several fermentation negative yeasts**

Growth data for fermentation negative yeast species and some additional fermentation competent species (latter denoted by *) in the presence of sorbic acid (SA; *R. glutinis* at 0.275 mM SA; *R. mucilaginosa,* 0.3 mM; P. *laurentii,* 0.4 mM; *C. magnus,* 0.1 mM; *Z. bailii*, 2 mM; *B. bruxellensis*, 2.5 mM) are represented by OD_600_ as a percentage of no-sorbic acid controls in 2% (black) or 0.1% (pink) glucose. Points represent means from three biological replicates, error bars (shown where larger than the dimensions of the symbols) represent SEM.

**Figure S4 – Effect of carbon source level on phenotype with sorbic acid (SA) depends on the carbon source**

Growth curves for *S. cerevisiae* W303 grown with glucose (**A**) or galactose (**B**) at 2% (black) or 0.1% (pink) are presented as OD_600_ values either without (open circles) or with (filled circles) 1 mM sorbic acid. **C.** Pressure generated by *S. cerevisiae* W303 grown at a starting inoculum of OD_600_ 0.2 in the absence or presence of 1 mM sorbic acid in either 2% or 0.1% galactose after 24 h static incubation at 24 °C, normalised by final OD_600_ readings. Points represent the means of three biological replicates; error bars represent SEM.

**Figure S5- Growth assays with sorbic acid for deletion mutants relevant to glucose sensing and repression.**

Growth assays of *S. cerevisiae* BY4743 (wild type) and isogenic deletions strains at 2% and 0.1% glucose with or without 0.75 mM sorbic acid. Points represent means from three biological replicates, error bars (shown where larger than the dimensions of the symbols) represent SEM. Results are summarised in Figure 5 of the main manuscript.
